# Chemokine expression in sera of patients with microscopic polyangiitis and granulomatosis with polyangiitis

**DOI:** 10.1038/s41598-024-59484-8

**Published:** 2024-04-15

**Authors:** Ji Eun Lee, Taejun Yoon, Sang-Won Lee, Sung Soo Ahn

**Affiliations:** 1 Department of Internal Medicine, Yonsei University College of Medicine, Seoul, Republic of Korea; 2 Department of Medical Science, College of Medicine, BK21 Plus Project, Yonsei University, Seoul, Republic of Korea; 3 Division of Rheumatology, Department of Internal Medicine, Yonsei University College of Medicine, 50-1 Yonsei-ro, Seodaemun-gu, Seoul, 03722 Republic of Korea; 4 Institute for Immunology and Immunological Diseases, Yonsei University College of Medicine, Seoul, Republic of Korea; 5 Division of Rheumatology, Department of Internal Medicine, Yonsei University College of Medicine, Yongin Severance Hospital, 363 Dongbaekjukjeon-daero, Giheung-gu, Yongin-si, Gyeonggi-do 16995 Republic of Korea

**Keywords:** Chemokines, Microscopic polyangiitis, Granulomatosis with polyangiitis, Activity, Fractalkine, Biomarkers, Rheumatology

## Abstract

We evaluated chemokine expression and its correlation with disease activity in patients with microscopic polyangiitis (MPA) and granulomatosis with polyangiitis (GPA) (MPA/GPA). Serum CCL2, CCL4, CCL19, CXCL1, CXCL2, and CX3CL1 level in 80 patients were analysed using multiple enzyme-linked immunosorbent assays. Correlations between variables were investigated using Pearson’s correlation analysis, and receiver operator curve analysis was performed to identify optimal CX3CL1 values in determining active disease. Multivariate logistic regression analysis was done to evaluate predictors of active disease. CCL4 (r = 0.251, *p* = 0.025), CXCL1 (r = 0.270, *p* = 0.015), and CX3CL1 (r = 0.295, *p* = 0.008) significantly correlated with BVAS, while CX3CL1 was associated with five-factor score (r =  − 0.290, *p* = 0.009). Correlations were revealed between CCL2 and CCL4 (r = 0.267, *p* = 0.017), CCL4 and CXCL1 (r = 0.368, *p* < 0.001), CCL4 and CXCL2 (r = 0.436, *p* < 0.001), and CXCL1 and CXCL2 (r = 0.518, *p* < 0.001). Multivariate analysis revealed serum CX3CL1 levels > 2408.92 pg/mL could predict active disease (odds ratio, 27.401, *p* < 0.001). Serum chemokine levels of CCL4, CXCL1, and CX3CL1 showed association with disease activity and especially, CX3CL1 > 2408.92 pg/mL showed potential in predicting active MPA/GPA.

## Introduction

Anti-neutrophil cytoplasmic antibody (ANCA)-associated vasculitis (AAV) is a systemic necrotizing vasculitis that manifests as small vessel inflammation^1^. Microscopic polyangiitis (MPA), granulomatosis with polyangiitis (GPA), and eosinophilic granulomatosis with polyangiitis (EGPA) are three entities comprising AAV that are separated by the pattern of organs affected, laboratory features, and distinguishing pathological findings within inflamed tissues^2^. Compared to EGPA, which has a favourable clinical outcome, MPA and GPA (MPA/GPA) are potentially fatal diseases with higher rates of death and progression to end-stage renal disease^3^. As disease severity is an important determinant of patient outcomes in AAV, studies have attempted to explore novel biomarkers, with a special focus on assessing disease activity^4,5^. Traditionally, laboratory tests of myeloperoxidase (MPO) and proteinase 3 (PR3)-ANCAs are useful biomarkers for the diagnosis and assessment of prognosis in the context of MPA/GPA^6^, but also with limitations that its titres alone are not sufficient to confirm diagnosis or monitor therapeutic response during follow‐up of patients^7^. In the meanwhile, the Birmingham Vasculitis Activity Score (BVAS) 3.0, which is composed of 56 items with a maximum score of 63, is a clinical measure for objectively describing ongoing inflammation in AAV^8^. Despite the significance of the BVAS, its implementation in clinical settings has limitations arising from the considerable time required for its assessment. Furthermore, challenges regarding the reproducibility and repeatability of the BVAS scores may emerge when evaluated by assessors with insufficient expertise.

Chemokines are small secreted substances first discovered in 1977^9^ that have molecular weights ranging between 8 and 10 kDA. Based on their biological behaviour and structure, chemokines are categorized as either possessing a homeostatic and inflammatory role or as CC, CXC, C, and CX3C chemokines according to their distinct cysteine residues^10^. Chemokines were initially described as chemoattractants that induce cell migration to the location of tissue injury and are responsible for maintaining host immunity against infections^11^. However, subsequent studies have demonstrated that chemokines exert a broad function in immune system homeostasis and act as critical regulators of inflammatory responses^12^. Indeed, accumulating evidence demonstrate that chemokines plays an important role in the pathophysiology of rheumatoid arthritis (RA) and systemic lupus erythematosus (SLE), which are representative autoimmune diseases (AIDs)^13–16^. In this context, studies have focused on evaluating chemokine levels and clinical features in AIDs such as RA, SLE, and ankylosing spondylitis (AS). Previous publications by Pandya et al. and Wang et al. identified that circulating CXCL10 levels are a disease activity marker in patients with untreated early RA and AS^17,18^. Meanwhile, several chemokines were revealed as potential markers of disease severity in SLE and predicted kidney involvement, implying that chemokines could be biological markers reflecting inflammation at the systemic and local levels in AIDs^19,20^. Similarly, there are investigations that assessed the role of chemokines expression in the circulation in AAV and its subtypes, showing association with clinical phenotypes or disease status^21–31^. Considering the increasing comprehension of chemokines in the development of AIDs, there is a necessity for enhanced insight into chemokines among patients with MPA/GPA; however, few studies have been conducted to date. Therefore, the present study sought to investigate expression of chemokines attracting monocytes/macrophages and T cells in the sera of patients with MPA/GPA, which were enrolled in a prospective cohort of AAV and to evaluate its correlation with disease activity.

## Methods

### Patients, blood samples, and clinical and laboratory data collection

Overall, this was a pilot study that included 80 patients with MPA and GPA from the Severance Hospital anti-neutrophil cytoplasmic antibody (ANCA)-associated VasculitidEs (SHAVE) cohort with available serum samples selected. The SHAVE cohort is an observational cohort comprising patients classified as having AAV according to the 2007 European Medicine Agency and the 2012 Chapel Hill Consensus Conference definitions^2,32^. Among patients included in the SHAVE cohort, clinical data and blood samples of patients with AAV were collected after obtaining consent^33,34^. For patients enrolled in this study, written informed consent was obtained from all subjects.

Clinical data of patients included age, sex, disease duration (from initial diagnosis to blood sample collection), BVAS (version 3), five-factor score (FFS) 2009, vasculitis damage index (VDI), and ANCA positivity when blood samples were obtained^8,35,36^, Patterns of organ involvement in patients were categorized according to the presence or absence of ear, nose, throat, nervous, renal, gastrointestinal, and cardiac features, as described in the BVAS. Laboratory test results were queried on the same date as blood collection and comprised the white blood cell (WBC) count, neutrophil count, erythrocyte sedimentation rate (ESR), and blood urea nitrogen (BUN), creatinine, aspartate aminotransferase (AST), alanine aminotransferase (ALT), haemoglobin, C-reactive protein (CRP), albumin levels.

### Chemokine level assessment in patient sera and active disease

Stored patient samples were used to evaluate chemokine levels in sera of patients with MPA/GPA. Levels of six chemokines—CCL19, CCL2, CCL4, CX3CL1, CXCL1, and CXCL2—were evaluated using multiplex ELISA (Abcam, Cambridge, UK) according to the manufacturer’s instructions. Active disease was principally defined according to the cut-off of BVAS ≥ 12, as previously described^37^. In addition, in a separate analysis of comparing the expression of chemokine levels according to disease status, we also applied the EULAR (European League Against Rheumatism) definition to define patients as having active disease and remission^38^.

### Statistical analyses

Continuous data are presented as medians (interquartile ranges), and categorical variables are presented as numbers (percentages). Differences in variables were evaluated using the Mann–Whitney U test and Kruskal–Wallis test for continuous variables and the chi-square test or Fisher’s exact test, as indicated. The interrelationship between continuous variables was assessed using Pearson’s correlation analysis, and receiver operator curve (ROC) analysis using the Youden index was used to derive the optimal CX3CL1 cut-off for predicting active disease. Multivariate logistic regression analysis using a forward entry method, including variables of statistical significance in the univariate analysis, was conducted to confirm the association between CX3CL1 and disease activity. In all statistical analyses, a two-tailed *p*-value of < 0.05 was considered statistically significant using MedCalc statistical software version 22 (MedCalc Software Ltd., Ostend, Belgium).

### Ethical approval

This study was performed in accordance with the principles set forth in the 1964 Helsinki Declaration and its later amendments and comparable standards and was approved by the Institutional Review Board of Severance Hospital (4-2016-0901).

## Results

### Characteristics of patients with MPA and GPA

Table 1 shows the clinical characteristics of the patients included in this study. Compared with patients with GPA, those diagnosed with MPA were older, had a shorter disease duration, and had higher BVAS and FFS. ENT involvement was more common in patients with GPA, whereas renal involvement was frequently observed in patients with MPA. In regard to laboratory tests, patients with MPA had lower haemoglobin and albumin levels than those in the GPA group. Concerning the expression of chemokines in patient sera, CX3CL1 expression was significantly higher in patients with MPA than in those with GPA; however, there was no difference in the expression of other chemokines according to patient diagnosis (Table 1). On the other hand, when we categorized the patients into active and inactive disease, 48 and 32 were included in the active and inactive disease group, respectively. Patients with active disease more frequently demonstrated renal manifestation compared to those without and the majority of laboratory variables investigated revealed significant differences between the groups, with an exception of AST and ALT (Table 2).

**Table 1:** Clinical characteristics of patients with MPA and GPA.

	MPA (n = 47)	GPA (n = 33)	*p*-value
Age, years	69.0 (58.0–73.8)	65.0 (49.8–70.5)	0.036
Female sex, n (%)	27 (57.4)	19 (57.6)	0.991
Disease duration < 3 month, n (%)	41 (87.2)	17 (51.5)	< 0.001
BVAS	16.0 (12.0–19.0)	6.0 (2.8–17.5)	< 0.001
FFS	2.0 (1.0–2.0)	1.0 (0.8–2.0)	0.005
VDI	3.0 (2.0–4.0)	3.0 (2.0–4.0)	0.880
ANCA positivity (%)	42 (89.4)	28 (84.8)	0.550
Patterns of organ involvement, n (%)			
ENT	10 (21.3)	17 (51.5)	0.005
Nervous	12 (25.5)	9 (27.3)	0.863
Renal	40 (85.1)	11 (33.3)	< 0.001
Gastrointestinal	1 (2.1)	0 (0.0)	0.999
Cardiac	4 (8.5)	2 (6.1)	0.999
Laboratory test results			
WBC count (/mm^3^)	9.2 (6.1–12.9)	7.8 (6.3–12.4)	0.494
Neutrophil (/mm^3^)	7.1 (4.6–10.5)	5.3 (3.7–10.5)	0.470
Hemoglobin (g/dL)	9.6 (8.6–11.5)	11.9 (10.2–13.5)	0.006
CRP (mg/L)	9.8 (1.4–84.9)	5.0 (1.1–30.4)	0.164
ESR (mm/hr)	64.0 (25.0–119.5)	37.0 (12.0–77.3)	0.061
BUN (mg/dL)	24.4 (18.7–37.9)	19.5 (13.7–27.4)	0.086
Creatinine (mg/dL)	1.3 (0.7–2.5)	0.9 (0.6–2.0)	0.276
AST (IU/L)	19.0 (14.3–23.5)	19.0 (13.8–27.5)	0.899
ALT (IU/L)	14.0 (9.0–21.5)	15.0 (11.8–27.3)	0.225
Albumin (g/dL)	3.2 (2.6–3.7)	4.1 (3.5–4.4)	< 0.001
Chemokine levels			
CCL19 (pg/mL)	192.8 (78.2–409.9)	114.5 (63.6–222.0)	0.132
CCL2 (pg/mL)	287.2 (194.8–376.5)	341.0 (228.3–414.0)	0.423
CCL4 (pg/mL)	408.9 (307.8–528.1)	395.9 (321.2–447.9)	0.541
CX3CL1 (pg/mL)	2780.3 (2350.1–3630.2)	1770.7 (1367.8–2570.6)	0.002
CXCL1 (pg/mL)	170.7 (94.6–351.5)	119.4 (65.7–195.1)	0.031
CXCL2 (pg/mL)	519.4 (350.3–966.0)	536.0 (325.4–912.4)	0.841

Values are presented as median (interquartile range) or n (%).

MPA: microscopic polyangiitis, GPA: granulomatosis with polyangiitis, BVAS: Birmingham vasculitis activity score, FFS: five-factor score, VDI: vasculitis damage index, ANCA: anti-neutrophil cytoplasmic antibody, ENT: ear, nose, and throat, WBC: white blood cells, CRP: C-reactive protein, ESR: erythrocyte sedimentation rate, BUN: blood urea nitrogen, AST: aspartate aminotransferase, ALT: alanine aminotransferase.

**Table 2:** Comparison of clinical and serological characteristics in patients with active and inactive disease.

	Active disease (n = 48)	Inactive disease (n = 32)	*p*-value
Age, years	69.0 (58.0–73.5)	61.0 (52.5–70.5)	0.059
Female sex, n (%)	26 (54.2)	20 (62.5)	0.463
Disease duration < 3 month, n (%)	44 (91.7)	14 (43.8)	< 0.001
AAV subgroup			< 0.001
MPA	37 (77.1)	10 (31.3)	
GPA	11 (22.9)	22 (68.8)	
BVAS	18.0 (14.0–21.0)	5.5 (2.5–8.0)	< 0.001
FFS	2.0 (1.5–2.0)	1.0 (1.0–2.0)	< 0.001
VDI	3.0 (2.0–4.0)	2.0 (2.0–3.5)	0.025
ANCA positivity (%)	45 (93.8)	25 (78.1)	0.080
Patterns of organ involvement, n (%)			
ENT	16 (33.3)	11 (34.4)	0.924
Nervous	14 (29.2)	7 (21.9)	0.471
Renal	44 (91.7)	7 (21.9)	< 0.001
Gastrointestinal	1 (2.1)	0 (0.0)	0.999
Cardiac	6 (12.5)	0 (0.0)	0.076
Laboratory test results			
WBC count (/mm^3^)	10.5 (7.8–14.1)	7.2 (5.3–9.6)	0.002
Neutrophil (/mm^3^)	7.9 (5.5–11.2)	4.9 (3.3–7.1)	0.005
Hemoglobin (g/dL)	9.3 (8.5–10.8)	12.8 (10.9–14.1)	< 0.001
CRP (mg/L)	19.7 (3.7–92.3)	1.3 (0.6–7.6)	< 0.001
ESR (mm/hr)	75.5 (32.0–119.5)	23.0 (12.0–56.0)	< 0.001
BUN (mg/dL)	28.3 (19.2–45.9)	16.7 (13.5–24.9)	0.001
Creatinine (mg/dL)	1.7 (0.8–3.2)	0.7 (0.6–1.2)	0.001
AST (IU/L)	19.5 (14.0–24.0)	18.5 (14.0–24.5)	0.922
ALT (IU/L)	12.5 (9.0–22.0)	18.0 (12.0–26.0)	0.164
Albumin (g/dL)	3.1 (2.7–3.7)	4.2 (3.6–4.4)	< 0.001

Values are presented as median (interquartile range) or n (%).

MPA: microscopic polyangiitis, GPA: granulomatosis with polyangiitis, BVAS: Birmingham vasculitis activity score, FFS: five-factor score, VDI: vasculitis damage index, ANCA: anti-neutrophil cytoplasmic antibody, ENT: ear, nose, and throat, WBC: white blood cells, CRP: C-reactive protein, ESR: erythrocyte sedimentation rate, BUN: blood urea nitrogen, AST: aspartate aminotransferase, ALT: alanine aminotransferase.

**Figure 1:**
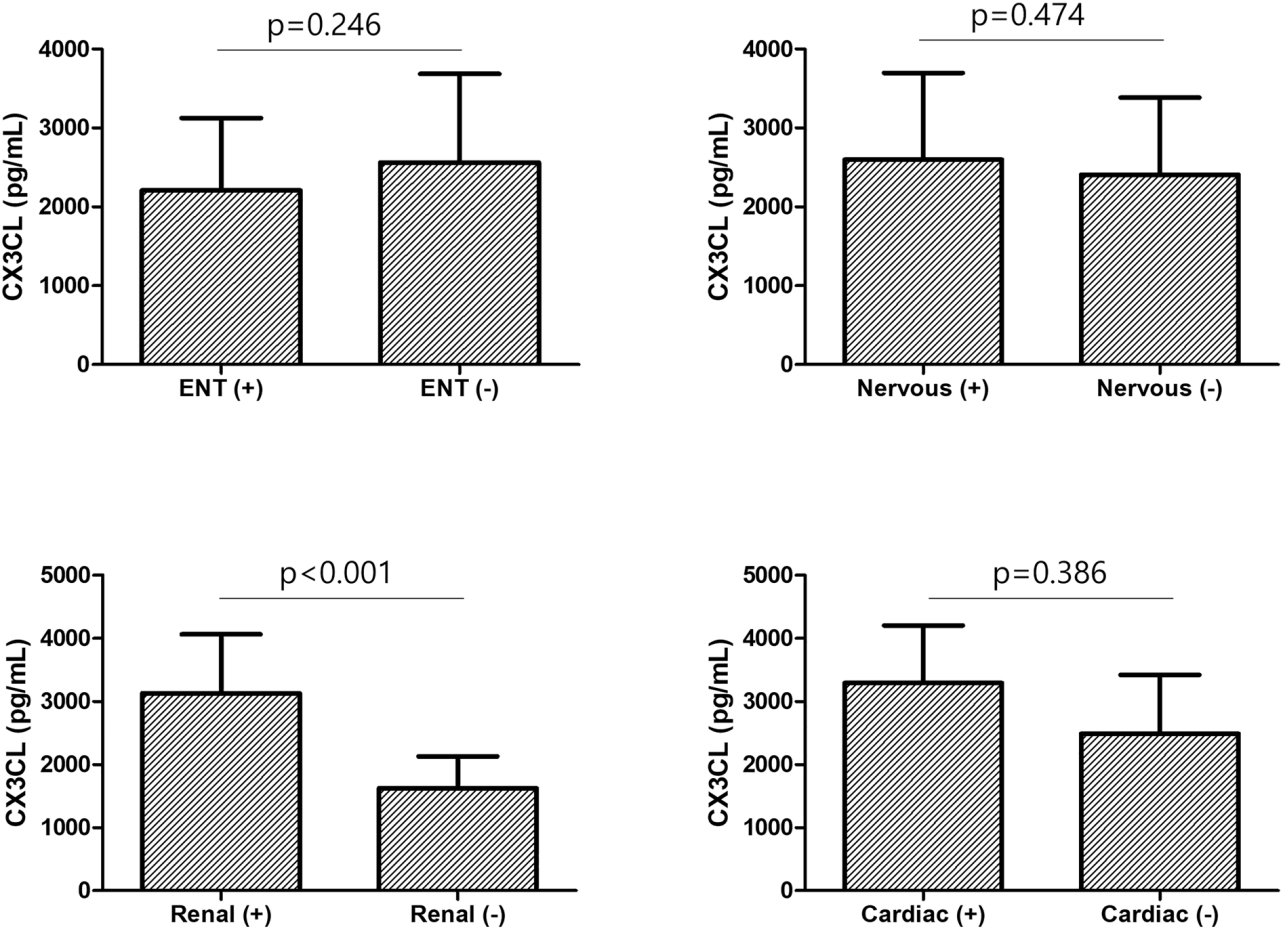
Serum CX3CL1 levels according to organ involvement. Patients with renal involvement showed significantly higher CX3CL1 levels than those without renal involvement. There were no differences in CX3CL1 levels according to the presence or absence of ENT, nervous system, or cardiac involvement. ENT, ear, nose, and throat.

Next, when the patients were divided into active and inactive disease and active disease and remission according to the BVAS cut-off of 12 and EULAR definition, respectively, the expression of CCL2 was lower and CX3CL1 was higher in patients with BVAS ≥ 12 than those with BVAS < 12, whereas the difference was only noted for CCL2 in patients with active disease and remission based on the EULAR definition. Moreover, on categorizing patients into the disease duration of 3 months and ANCA groups, the expression of chemokine CX3CL1 was significantly higher in the disease duration < 3 months group. In contrast, CXCL1 expression was higher in patients with a disease duration of < 3 months and in those with ANCA positivity (Table 3). A comparison of CX3CL1 levels according to the pattern of organ involvement indicated that patients with renal involvement had significantly higher CX3CL1 levels than those without (*p* < 0.001) (Fig. 1).

**Table 3:** Comparison of chemokines levels according to disease status, disease duration, and ANCA positivity.

Chemokines	Disease status (by BVAS ≥ 12)			Disease status (by EULAR definition)		
	Active disease	Inactive disease	*p*-value	Active disease	Remission	*p*-value
CCL19 (pg/mL)	204.5 (66.2–366.4)	124.2 (70.1–219.1)	0.235	175.7 (66.2–317.1)	110.3 (78.9–234.7)	0.464
CCL2 (pg/mL)	252.3 (191.0–347.5)	356.6 (297.8–430.7)	0.004	256.0 (185.3–351.1)	392.1 (325.6–500.7)	< 0.001
CCL4 (pg/mL)	408.9 (312.3–537.3)	392.6 (321.2–450.7)	0.420	395.9 (303.4–528.1)	421.3 (321.2–456.4)	0.813
CX3CL1 (pg/mL)	2970.4 (2485.8–3745.6)	1722.7 (1323.7–2189.0)	< 0.001	2708.4 (1770.7–3699.8)	2018.1 (1623.9–2561.3)	0.086
CXCL1 (pg/mL)	166.3 (90.8–309.0)	125.3 (77.1–226.5)	0.193	175.0 (90.8–330.7)	112.7 (90.8–152.3)	0.161
CXCL2 (pg/mL)	452.8 (344.8–1048.0)	573.0 (358.6–898.7)	0.637	550.2 (349.9–1047.3)	499.3 (332.9–729.1)	0.583

Chemokines	Disease duration			ANCA positivity		
	Disease duration < 3 month	Disease duration ≥ 3 month	*p*-value	ANCA positive	ANCA negative	*p*-value
CCL19 (pg/mL)	170.5 (64.3–320.7)	124.2 (81.7–225.3)	0.647	151.5 (66.2–310.7)	86.3 (61.1–261.7)	0.432
CCL2 (pg/mL)	256.0 (190.7–351.1)	392.1 (325.6–472.7)	< 0.001	298.3 (206.1–416.1)	322.7 (191.4–361.4)	0.954
CCL4 (pg/mL)	402.4 (303.4–524.1)	401.9 (321.2–456.4)	0.500	408.9 (321.2–528.1)	349.4 (321.2–456.4)	0.403
CX3CL1 (pg/mL)	2726.5 (1817.7–3699.8)	1863.5 (1466.9–2485.8)	0.018	2485.8 (1722.7–3669.1)	2634.9 (1172.4–3322.3)	0.626
CXCL1 (pg/mL)	179.2 (90.8–330.7)	106.1 (82.0–152.3)	0.021	161.8 (90.8–292.5)	98.4 (46.3–152.3)	0.048
CXCL2 (pg/mL)	550.2 (349.9–1047.3)	512.3 (332.9–729.1)	0.659	543.4 (349.9–969.1)	469.1 (332.9–572.4)	0.485

ANCA: anti-neutrophil cytoplasmic antibody, BVAS: Birmingham vasculitis activity score, EULAR: European League Against Rheumatism.

### Correlation between chemokines and clinical parameters

Correlation analysis revealed significant associations between CCL2 and CCL4 (r = 0.267, *p* = 0.017), CCL4 and CXCL1 (r = 0.368, *p* < 0.001), CCL4 and CXCL2 (r = 0.436, *p* < 0.001), and between CXCL1 and CXCL2 (r = 0.518, *p* < 0.001). CX3CL1 expression was not significantly associated with any of the other evaluated chemokines (Table 4).

**Table 4:**
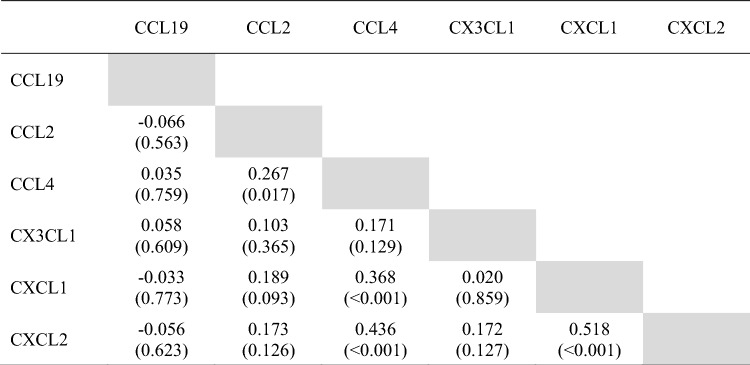
Inter-correlation between the evaluated chemokines.

Values indicate correlation coefficient and *p*-values.

Additionally, there was a significant correlation between CCL4, CX3CL1, CXCL1, and BVAS a(r = 0.251, 0.295, and 0.270, respectively), whereas CX3CL1 correlated only with FFS (r = 0.0290, *p* = 0.009). However, there was no relationship between the VDI and the chemokines investigated (Table 5).

**Table 5:** Correlation of chemokines and clinical parameters.

	CCL19	CCL2	CCL4	CX3CL1	CXCL1	CXCL2
BVAS	0.152 (0.179)	− 0.026 (0.820)	0.251 (0.025)	0.295 (0.008)	0.270 (0.015)	0.033 (0.768)
FFS	0.067 (0.556)	− 0.012 (0.919)	0.077 (0.498)	0.290 (0.009)	0.024 (0.830)	− 0.087 (0.445)
VDI	0.184 (0.102)	0.074 (0.513)	− 0.040 (0.724)	0.126 (0.264)	0.023 (0.843)	− 0.204 (0.070)

BVAS: Birmingham vasculitis activity score, FFS: five-factor score, VDI: vasculitis damage index.

### Prediction of disease status according to serum CX3CL1 levels

**Figure 2:**
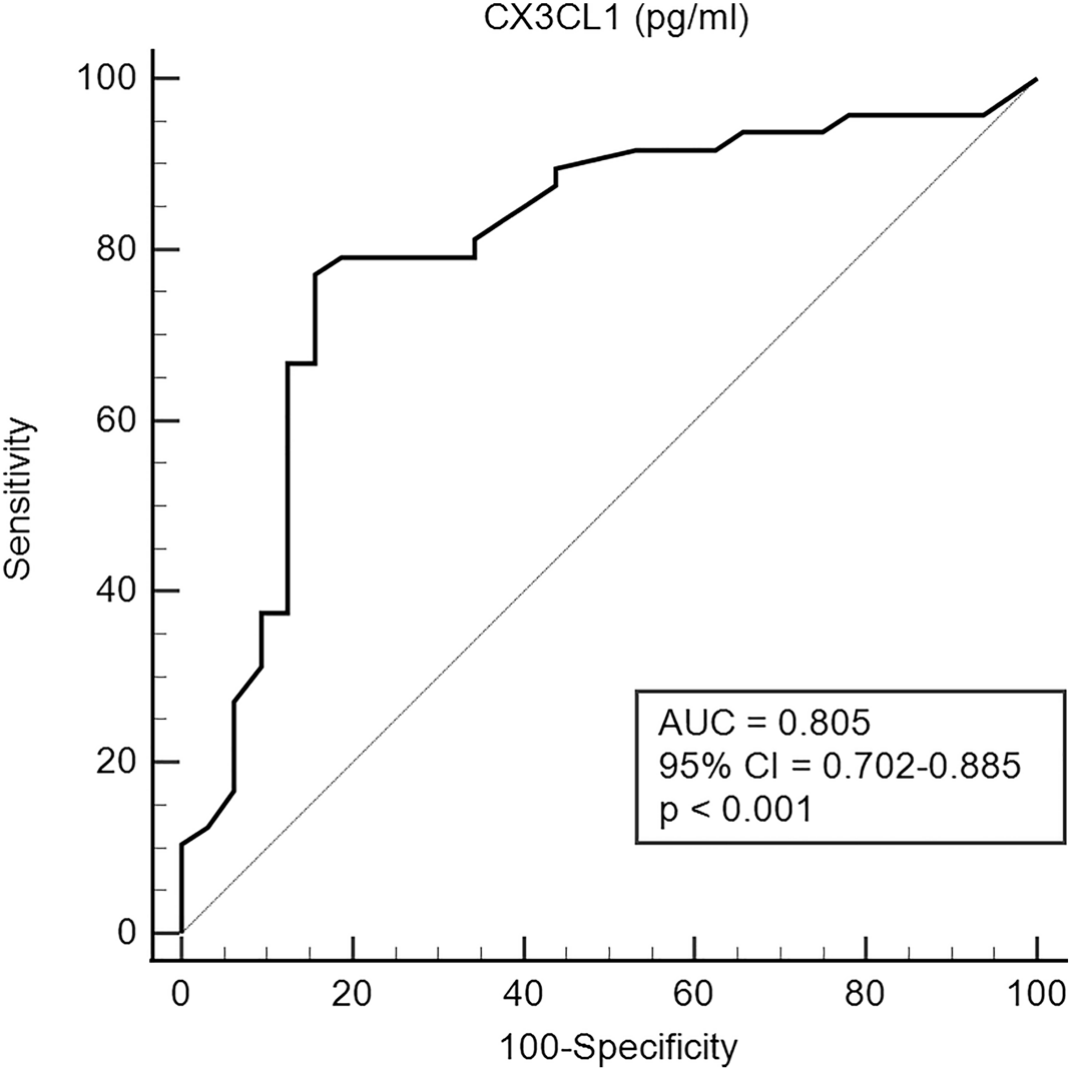
Receiver operator curve analysis for the ideal cut-off value of CX3CL1 in predicting active disease. The derived cut-off value for CX3CL1 in determining active disease was 2408.92 pg/mL. AUC, area under the curve.

ROC analysis indicated that the ideal cut-off value of CX3CL1 for discriminating between active and inactive disease defined with the BVAS cut-off was 2408.92 pg/mL (area under the ROC, 0.805; 95% confidence interval [CI], 0.702–0.885, *p* < 0.001) (Fig. 2). In univariate logistic regression, WBC count, ESR, and haemoglobin, CRP, BUN, creatinine, albumin, and CX3CL1 levels were associated with the presence of active disease. A CX3CL1 level > 2408.92 pg/mL conferred approximately 18 times greater risk of possessing active disease (odds ratio [OR], 18.164; 95% CI 5.650–58.392, *p* < 0.001). In the multivariable analysis using CX3CL1 as a continuous variable, WBC count, albumin level, and CX3CL1 levels remained independent predictors of active disease, and a CX3CL1 level > 2408.92 pg/mL showed that the risk of active AAV increased by nearly 27 times (OR, 27.401; 95% CI 5.773–130.052, *p* < 0.001), along with the WBC count (OR, 1.241; 95% CI 1.033–1.492, *p* = 0.021) and albumin level (OR, 0.315; 95% CI 0.114–0.870, *p* = 0.026) (Table 6).

**Table 6:** Logistic regression analysis for the prediction of active disease.

	Univariable analysis			Multivariable analysis*			Multivariable analysis**		
	OR	95% CI	*p*-value	OR	95% CI	*p*-value	OR	95% CI	*p*-value
Age	1.021	0.989–1.054	0.209						
Sex	0.709	0.284–1.768	0.461						
ANCA positivity	4.200	0.997–17.695	0.051						
WBC count	1.190	1.053–1.345	0.005	1.171	1.004–1.366	0.044	1.241	1.033–1.492	0.021
Neutrophil	1.035	0.955–1.122	0.404						
Hemoglobin	0.602	0.470–0.771	< 0.001						
CRP	1.017	1.004–1.030	0.011						
ESR	1.023	1.010–1.036	< 0.001						
BUN	1.051	1.013–1.089	0.008						
Creatinine	1.953	1.198–3.183	0.007						
AST	0.999	0.960–1.039	0.945						
ALT	1.001	0.980–1.022	0.963						
Albumin	0.146	0.060–0.355	< 0.001	0.286	0.109–0.746	0.011	0.315	0.114–0.870	0.026
CCL19	1.000	0.999–1.001	0.509						
CCL2	0.999	0.998–1.001	0.393						
CCL4	1.002	0.999–1.004	0.183						
CX3CL1*	1.002	1.001–1.002	< 0.001	1.001	1.000–1.002	0.003			
CXCL1	1.002	0.999–1.005	0.125						
CXCL2	1.000	0.999–1.001	0.939						
CX3CL1 2408.92 > pg/mL**	18.164	5.650–58.392	< 0.001				27.401	5.773–130.052	< 0.001

*CX3CL1 included in the multivariate analysis.

**CX3CL1 2408.92 > pg/mL included in the multivariable analysis.

ANCA: anti-neutrophil cytoplasmic antibody, WBC: white blood cell, CRP: C-reactive protein, ESR: erythrocyte sedimentation rate, BUN: blood urea nitrogen, AST: aspartate aminotransferase, ALT: alanine aminotransferase.

## Discussion

Chemokines play a crucial role in coordinating immune cell migration towards inflamed tissues, leading to their activation^39^. Consequently, chemokines produced by various cell types in response to pro-inflammatory signals serve as key mediators that bridge the innate and adaptive immune systems.^40^ In this study, we observed a difference in the expression of CX3CL1 and CXCL1 in sera of patients with MPA and GPA. Additionally, CCL4, CX3CL1, and CXCL1 were significantly associated with disease activity as assessed using the BVAS, and only CX3CL1 correlated with FFS. The relationship between chemokines and organ damage, estimated using the VDI, was not evident. Notably, a CX3CL1 level > 2408.92 pg/mL was an independent predictive factor of active disease even when laboratory tests that are generally performed during routine patient care were taken into account, indicating that CX3CL1 could serve as a potential biological marker for the evaluation of disease activity in MPA/GPA.

Several factors may indicate an association between chemokine expression and disease activity in MPA/GPA. Heightened inflammation in MPA/GPA can be reflected in various factors, including chemokine upregulation. An increase in chemokine levels can occur because of enhanced cytokine production and polarization of helper T and B cell subsets, corresponding to greater levels of inflammation. A characteristic feature of MPA/GPA is an imbalance in cytokine production, with cytokines, such as tumour necrosis factor-alpha, interleukin (IL)-6, and IL-17, implicated in disease pathogenesis^41^. These cytokines stimulate chemokine production, thereby promoting immune cell recruitment and inflammation^42^. Additionally, the expansion of Th1 and Th17 helper T cell subsets, which play a pivotal role in MPA/GPA, leads to the release of inflammatory cytokines that further induce chemokine production, thereby amplifying pro-inflammatory signalling in the disease^43^. Furthermore, B cells specifically contribute to MPA/GPA by producing ANCA antibodies^44^. Abnormal activation and differentiation of B cells can enhance disease activity by overexpressing pathogenic antibodies and cytokines such as B cell-activating factors, thereby promoting chemokine production and exacerbating inflammation^45^. Lastly, activated monocytes and macrophages at the site of inflammation also contribute to the perpetuation of inflammatory cycle and damage in MPA/GPA^46^. Conversely, increased chemokine expression in MPA/GPA intrinsically influences the recruitment and infiltration of specific leukocyte subsets into affected tissues^47^. As these immune cells contribute to inflammatory processes within blood vessels, this could result in tissue injury in MPA/GPA.

To date, various studies have reported the expression of circulating chemokines in patients with AAV. Recent publications have demonstrated that CXCL9 and CXCL16 are increased in AAV compared to controls^23,25^. In addition, a distinct chemokine levels in the circulation was also identified in PR3-ANCA AAV according to organ involvement^21^, and a prognostic role of CCL18 in ANCA-associated glomerulonephritis has been also described^22^, with the disease activity status predicting potential of CCR8, CXCL2, CXCL13, CCL5, CCL20, CCL22 suggested in AAV^24,26–29^. On the other hand, investigation of CX3CL1 in patients with MPA and GPA revealed increased level in the periphery compared to controls, with relationship present with BVAS and active disease, respectively^30,31^. Nevertheless, due to significant discrepancies in defining AAV and disease status, differences in evaluated AAV subtypes and organ involvement, and the restricted patient sample size, the clinical implications of assessing chemokines in the peripheral blood of patients with AAV is still unclear which deserve better understanding.

This study revealed that of the six chemokines examined, only CX3CL1 levels were independently associated with disease activity in MPA/GPA. CX3CL1, also referred to as fractalkine, may serve as an indicator of ongoing inflammation in MPA/GPA because it may potentially stimulate the activation and recruitment of neutrophils, which play a crucial role in the development of MPA/GPA^48^. Elevated levels of CX3CL1 augment the attachment of neutrophils to endothelial cells, facilitating their movement into inflamed tissues and causing tissue damage. Additionally, CX3CL1 signalling through its receptor CX3CR1 can initiate intracellular signalling pathways in immune cells, leading to the generation of pro-inflammatory cytokines and chemokines and contributing to the observed inflammatory response^49^. Furthermore, CX3CL1 influences T cell migration, activation, and production of T cell-related cytokines, potentially contributing to the immune dysregulation observed in MPA/GPA^50^. Alternatively, increased levels of CX3CL1, which is expressed in endothelial cells, may indicate endothelial activation^51^. Activated endothelial cells upregulate adhesion molecules, facilitate leukocyte recruitment, and perpetuate inflammation in patients with MPA/GPA. Supporting these hypotheses, previous studies have indicated the involvement of CX3CL1 in various type of vasculopathies^30,31,52,53^; of note, our findings showed that patients with MPA had higher levels of CX3CL1 compared to those with GPA. Even though the elevated BVAS in patients with MPA compared to those with GPA may account for this observation, given that previous research indicated the involvement of the CX3CL1-CX3CR1 axis in organ damage in MPO-ANCA-associated vasculitis^54^, it is plausible that CX3CL1 may play a greater role in promoting inflammation in MPA, where MPO-ANCA and renal involvement is more frequently observed.

The results of our study suggest that CX3CL1 could potentially be used as a biomarker to assess disease activity in MPA/GPA. Monitoring CX3CL1 levels could offer healthcare providers useful information regarding the extent of the inflammatory response and severity of the disease. It is worth noting that in addition to laboratory measures, such as WBC count and albumin levels, a CX3CL1 level of 2408.92 pg/mL was a predictive factor for active disease status. Furthermore, the correlation between CX3CL1 and FFS indicates that CX3CL1 may also have prognostic implications as FFS is reportedly related to adverse clinical outcomes in patients with AAV^35^. Therefore, incorporating CX3CL1 testing into clinical practice may be beneficial for providing optimal care to patients with MPA/GPA, with increased attention required for those exhibiting higher CX3CL1 levels. Additionally, considering that blocking CX3CL1 has shown efficacy in reducing inflammation in autoimmune disorders and potential for treating RA, it is plausible that targeting CX3CL1 could also have beneficial effects in managing MPA/GPA^55^. However, owing to the limitations of this cross-sectional study, we were unable to address whether assessing CX3CL1 could be useful in monitoring treatment response and predicting disease-related outcomes. Further investigations are necessary to explore these aspects.

The limitations of this study included the fact that both incident and prevalent cases of MPA/GPA were included in the analysis, and adjustment for the treatment regimen could not be performed. Furthermore, while the present study showed that CX3CL1 might be a biomarker of AAV, additional longitudinal assessments are required to verify its advantage in the follow-up of patients with MPA/GPA. Finally, the pathogenic mechanism linking CX3CL1 to inflammation in MPA/GPA remains to be elucidated in future studies.

In conclusion, our study demonstrated that among serum chemokines, CX3CL1 correlated with disease activity and was independently associated with active disease in patients with MPA/GPA. Hence, our findings suggest that evaluating CX3CL1 levels could have clinical implications for predicting disease severity.

## Data Availability

The data that support the findings of this study are available from the corresponding author upon reasonable request.
